# Optimization and Comparative Evaluation of DNA Extraction Protocols for Arbuscular Mycorrhizal Fungi in Coffee Roots

**DOI:** 10.3390/mps9050153

**Published:** 2026-10-21

**Authors:** Lorena Jacqueline Gómez-Godínez, Alejandra Ortiz-Gasca, José Luis Aguirre-Noyola, José Martin Ruvalcaba-Gómez, Sergio De los Santos-Villalobos, Ramón Ignacio Arteaga-Garibay

**Affiliations:** 1 Centro Nacional de Recursos Genéticos, Instituto Nacional de Investigaciones Forestales, Agrícolas y Pecuarias, Boulevard de la Biodiversidad 2498, Rancho las Cruces, Tepatitlán de Morelos 47714, Jalisco, Mexico; gomez.lorena@inifap.gob.mx (L.J.G.-G.); aguirre.jose@inifap.gob.mx (J.L.A.-N.); ruvalcaba.josemartin@inifap.gob.mx (J.M.R.-G.); 2 Doctorado en Micología Aplicada, Centro de Investigación en Micología Aplicada, Universidad Veracruzana, Médicos No. 5, U.H. del Bosque, Xalapa 91010, Veracruz, Mexico; zs23000789@estudiantes.uv.mx; 3 Departamento de Ciencias Agronómicas y Veterinarias, Instituto Tecnológico de Sonora, 5 de febrero 818 Sur, Cd. Obregón 85000, Sonora, Mexico; sergio.delossantos@itson.edu.mx

**Keywords:** arbuscular mycorrhizal fungi, DNA extraction, *Coffea arabica*

## Abstract

Molecular studies of arbuscular mycorrhizal fungi (AMF) in coffee root require DNA extracts that are sufficiently pure for PCR amplification. *Coffea arabica* roots remains challenging because of the abundance of phenolic compounds, polysaccharides, and other inhibitors. This study compared four DNA extraction protocols, including two CTAB-based phenol–chloroform methods, a commercial extraction kit, and a modified commercial kit incorporating mechanical disruption, proteinase K predigestion, and β-mercaptoethanol treatment. Quality of total DNA extracts was assessed using NanoDrop spectrophotometry (A260/A280 and A260/A230 ratios), agarose gel electrophoresis, and PCR amplification of the AMF SSU (18S rDNA) region using AMV4.5NF/AMDGR primers. Conventional phenol–chloroform methods yielded high DNA concentrations but poor purity, indicating substantial co-extraction of inhibitory compounds. The commercial kit without predigestion yielded lower spectrophotometric purity but still produced successful AMF-targeted PCR amplification. In contrast, the modified protocol yielded the highest spectrophotometric purity ratios while retaining successful amplification across all samples. These results indicate that incorporation of the predigestion step was associated with higher post-extraction spectrophotometric ratios without compromising successful AMF-targeted PCR amplification. The optimized protocol provides a reproducible method for obtaining PCR-quality DNA from coffee roots and represents a methodological contribution to molecular studies of the diversity of AMF and other root-associated microorganisms in coffee agroecosystems.

## 1. Introduction

In coffee agroecosystems, arbuscular mycorrhizal fungi (AMF) actively participate in rhizosphere dynamics, playing a crucial role in processes such as phosphorus acquisition, nitrogen redistribution, and modulation of stress responses [1,2]. These multifactorial functions have positioned AMF as strategic components in the development of more sustainable and resilient agricultural systems [3].

The characterization of AMF communities remains a technically challenging [4]. Morphological identification is commonly based on spores recovered from soil, but this approach may not accurately represent the taxa colonizing roots at the time of sampling and often provides limited taxonomic resolution [5,6]. In this context, molecular tools, such as polymerase chain reaction (PCR) and massive sequencing techniques, have become established as essential methods for analyzing the diversity and composition of AMF [7] directly in root tissues [8].

However, the reliability of these approaches depends critically on the quality and purity of the extracted total DNA, making the extraction step a key step in the methodological workflow [9]. Various studies have developed different approaches for AMF DNA extraction from root tissues. Among the most widely used are classic protocols based on detergents, such as cetyltrimethylammonium bromide (CTAB) [10], which facilitate cell lysis and polysaccharide removal. These methods are often complemented by chemical modifications, such as the incorporation of reducing agents (e.g., β-mercaptoethanol) and compounds like polyvinylpyrrolidone (PVP) [11], which act as sequestering agents for polyphenols and tannins by forming hydrogen bonds, thereby improving the purity of the DNA obtained. Furthermore, the inclusion of enzymatic digestion steps using proteinase K to remove associated proteins is common [12]. Phenol–chloroform extraction can also improve the separation of nucleic acids from proteins and other cellular components, whereas commercial silica-column kits provide a more standardized workflow. Nevertheless, the performance of each method depends on the chemical composition and physical characteristics of the tissue being processed [13,14].

Despite advances, a large proportion of protocols have focused on soil matrices or general plant tissues, with little attention paid to particularly complex systems. Coffee roots constitute a particularly challenging system due to their high content of secondary metabolites, including polyphenols, tannins, alkaloids such as caffeine, and other highly reactive phenolic compounds [15,16]. These compounds can be co-extracted with nucleic acids during extraction, leading to DNA degradation, alterations in purity ratios (260/280 and 260/230), and, critically, inhibition of enzymatic reactions such as PCR. It has been documented that phenolic compounds can interact with DNA and proteins via hydrogen bonding and oxidation, forming complexes that interfere with DNA polymerase activity and reduce amplification efficiency [17,18,19].

Because AMF associated with roots represent the fungal fraction directly interacting with the host at the time of sampling [20], inefficient DNA extraction may lead to amplification failure or biased detection of AMF taxa, compromising the ecological interpretation of the results. In this context, it is necessary to develop and evaluate DNA extraction protocols specifically adapted to *coffee* roots.

Therefore, this study compared four procedures for extracting total DNA from *Coffea arabica* roots. The resulting extracts were evaluated according to DNA concentration, spectrophotometric purity, electrophoretic integrity, and their suitability for amplification of an AMF-targeted SSU 18S rDNA fragment. NanoDrop measurements were interpreted as estimates of total root-associated DNA and not as a direct quantification of AMF DNA.

Particular attention was given to determining whether predigestion with proteinase K and β-mercaptoethanol improved the performance of a commercial silica-column kit; this work aims to advance the standardization of molecular workflows for studying AMF in agroecosystems.

## 2. Sampling and Experimental Design

Sampling was conducted on Coffea arabica var. Garnica plants established in the field for more than 20 years, located at Finca Consolapan, municipality of Coatepec, Veracruz, Mexico (19°28′36.03″ N, 96°56′39.62″ W). As shown in Figure 1, nine *Coffea arabica* plants were selected and distributed among the experimental treatments established in the field. For each treatment, approximately 500 mg of fresh root was obtained from each plant; the fine roots were combined to generate a representative composite sample. This was done to reduce intra-treatment variability and to have sufficient root tissue available to evaluate the different extraction protocols. In total, four composite samples were obtained, each of which was subdivided for the application of the four DNA extraction methods.

The root material used in this study originated from a previous experiment in which arbuscular mycorrhizal colonization had been confirmed through root clearing and staining according to [21], followed by microscopic quantification using the gridline intersect method of [22]. However, colonization percentage was not considered an experimental variable in the present study because all extraction methods were applied to identical composite root samples.

Because the study aimed to compare the performance of different DNA extraction protocols and not to characterize the diversity of arbuscular mycorrhizal fungi (AMF) in the field, all samples were obtained from the same experimental site and processed under standardized conditions. This design minimized the biological variability associated with the origin of the samples and allowed for the evaluation of differences attributable solely to the extraction methods.

The collected roots were carefully cleaned in the field by removing excess soil with a dry brush, avoiding washing with water to minimize the loss of surface fungal DNA and reduce the risk of contamination. The samples were then placed in sterile Falcon tubes with silica gel beads for partial desiccation and preservation during shipping. Once in the laboratory, the samples were stored at 4 °C until processing for DNA extraction.

## 3. Procedure

### 3.1. Preparation of Root Samples

Estimated Time: 30–40 min

1. Select fine roots of Coffea arabica previously dried with silica gel.
2. Remove excess soil using a sterile, dry brush.
3. Weigh approximately 250 mg of root tissue per sample.
4. Place the sample in a sterile 1.5 mL conical tube.
5. Macerate the tissue with a pestle in liquid nitrogen until a fine powder is obtained. Because the grinding time depended on tissue characteristics, no fixed grinding time was established.

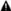
 **CRITICAL STEP**

Perform maceration quickly to avoid thawing the tissue, which can promote DNA degradation and oxidation of phenolic compounds.

### 3.2. Method 1: Phenol–Chloroform Extraction

Estimated time 3–4 h (plus overnight incubations)

#### 3.2.1. Lysis and Tissue Disruption

1. Place the previously macerated sample in a sterile 1.5 mL conical tube.
2. Add 350 µL of TE buffer prepared by mixing 50 mL of 10 mM Tris-HCl (pH 8.0) and 20 mL of 0.5 M EDTA (pH 8.0) stock solutions, followed by sterilization by autoclaving and storage at 4 °C, and 350 µL of preheated (65 °C) lysis buffer (10% CTAB, 1 M Tris-HCl pH 8.0, 0.5 M EDTA pH 8.0, and 2.5 M NaCl); the buffer was sterilized through autoclaving and stored at room temperature until use.
3. Incorporate approximately 200 mg of sterile glass or porcelain beads (0.1 mm).
4. Vortex vigorously for 30 min.

#### 3.2.2. Reduction in Phenolic Compounds and Lysis

1. Add 30 µL of β-mercaptoethanol (Sigma-Aldrich. Merck KGaA, Darmstadt, Germany) (final concentration: 4.1% *v*/*v*).
2. Incubate at 65 °C with shaking at 300 rpm in a thermoblock for 45 min.

#### 3.2.3. Protein Digestion

1. Add 6 µL of proteinase K (Sigma-Aldrich. Merck KGaA, Darmstadt, Germany), 20 mg/mL (final concentration 163 µg/mL).
2. Vortex.
3. Incubate at 56 °C for 1 h.
4. Gently mix every 10 min during incubation.

#### 3.2.4. Extraction with Phenol: Chloroform

1. Add 500 µL of phenol–chloroform-isoamyl alcohol (25:24:1), leaving enough space to close the tube; reduce the volume if necessary.
2. Vortex until homogeneous.
3. Incubate at 65 °C for 30 min.
4. Perform a thermal shock with liquid nitrogen (the tubes were immediately immersed in liquid nitrogen) for 30 min, without agitation.
5. Remove the tubes and centrifuge at 12,000 rpm for 10 min at room temperature.
6. Carefully collect the aqueous phase in a new tube.

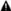
 **CRITICAL STEP**

Avoid transferring the interface to prevent contamination with proteins and organic compounds.

#### 3.2.5. Chloroform Washes

1. Add 500 µL of chloroform:isoamyl alcohol (24:1).
2. Mix by inversion for 2 min.
3. Centrifuge at 12,000 rpm for 10 min.
4. Recover the aqueous phase and transfer it to a new tube.
5. Repeat the wash 2–4 times.

#### 3.2.6. DNA Precipitation

1. Transfer the final aqueous phase to a clean tube.
2. Add 1 mL of cold isopropanol or cold absolute ethanol.
3. Mix gently by inversion.
4. Incubate at −20 °C overnight.
5. Centrifuge at 12,000 rpm for 10 min.
6. Discard the supernatant.
7. Wash the pellet with 1 mL of 70% ethanol.
8. Centrifuge at 12,000 rpm for 10 min.
9. Discard the supernatant.
10. Air-dry.

#### 3.2.7. DNA Resuspension

1. Resuspend the DNA in 35 µL of nuclease-free water.

### 3.3. Method 2: Phenol–Chloroform Extraction with Modification of the Lysis Buffer (PVP)

Estimated time 3–4 h (plus overnight incubations)

This method was performed following the procedure described in Section 3.2, with the following modification to the sample preparation and lysis buffer composition:

(a) Direct maceration in a mortar with liquid nitrogen.
  1. Following the procedure described in Section 3.2, freeze the root tissue with liquid nitrogen in a pre-chilled mortar.
  2. Macerate until a homogeneous fine powder is obtained.
(b) Modification of the lysis buffer (PVP).
  1. Prepare the lysis buffer by adding polyvinylpyrrolidone (PVP, 2% *w*/*v*) to the lysis buffer described in Section 3.2.1.
  2. Use this modified buffer in the lysis step (Step 4 of Section 3.2).
  3. Sample processing continues in the same manner as in Section 3.2.

### 3.4. Method 3: DNA Extraction Using the Quick-DNA™ Fecal/Soil Microbe Miniprep Kit (Zymo Research, Irvine, CA, USA; Cat. No. D6010)

Estimated time: 60–90 min.

1. Weigh 250 mg of root tissue previously ground in liquid nitrogen as described in Section 3.1 and transfer it to a ZR BashingBead™ Lysis Tube (Zymo Research, Irvine, CA, USA).
2. Add 750 µL of BashingBead™ Buffer (Zymo Research, Irvine, CA, USA) and tightly cap the tube.
3. Homogenize the sample at maximum speed for 30 min.
4. Centrifuge the ZR BashingBead™ Lysis Tube at ≥10,000× *g* for 1 min.
5. Transfer up to 400 µL of the supernatant to a Zymo-Spin™ III-F Filter (Zymo Research, Irvine, CA, USA) placed in a Collection Tube and centrifuge at 8000× *g* for 1 min. Discard the filter.
6. Add 1200 µL of Genomic Lysis Buffer previously supplemented with β-mercaptoethanol to a final concentration of 0.5% (*v*/*v*) to the filtrate and mix thoroughly.
7. Transfer 800 µL of the mixture to a Zymo-Spin™ IICR Column (Zymo Research, Irvine, CA, USA) placed in a Collection Tube and centrifuge at ≥10,000× *g* for 1 min. Discard the flow-through and repeat this step with the remaining mixture.
8. Transfer the column to a new Collection Tube, add 200 µL of DNA Pre-Wash Buffer, and centrifuge at ≥10,000× *g* for 1 min.
9. Add 500 µL of g-DNA Wash Buffer and centrifuge at ≥10,000× *g* for 1 min.
10. Transfer the column to a clean 1.5 mL microcentrifuge tube and add 50 µL of DNA Elution Buffer directly to the column matrix. Centrifuge at ≥10,000× *g* for 30 s to elute the DNA.
11. Prepare the Zymo-Spin™ IV-IR HRC Filter (Zymo Research, Irvine, CA, USA) by loosening the screw cap and removing the bottom tip. Place the filter in a clean Collection Tube and centrifuge at 8000× *g* for 3 min.
12. Transfer the eluted DNA to the prepared Zymo-Spin™ IV-IR HRC Filter placed in a clean 1.5 mL microcentrifuge tube and centrifuge at exactly 8000× *g* for 3 min.
13. Store the purified DNA at −20 °C until further analysis.

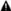
 **CRITICAL STEP**

Root samples may absorb all of the BashingBead buffer; add 375 µL and repeat the centrifugation cycle if necessary.

### 3.5. Method 4: DNA Extraction Using the Quick-DNA™ Fecal/Soil Microbe Miniprep Kit by Zymo Research with Predigestion Treatment

Estimated time: 2–3 h.

#### 3.5.1. Tissue Predigestion and Preparation

1. Grind the sample in a sterile mortar with liquid nitrogen until a homogeneous powder is obtained.
2. Add 2.3 mL of TE buffer (50:20) to the pulverized material.
3. Continue maceration until a homogeneous paste is obtained.
4. Transfer the sample to a 1.5 mL microcentrifuge tube. To facilitate transfer, cut 2–3 mm off the tip of a sterile micropipette.

#### 3.5.2. Enzymatic Digestion and Inhibitor Reduction

1. Add 6 µL of proteinase K,20 mg/mL (final concentration 51 µg/mL)and 30 µL of β-mercaptoethanol (final concentration 1.3%).
2. Vortex for 10 s.
3. Incubate at 56 °C for 60 min with gentle shaking (~300 rpm).
4. Subsequently incubate at 65 °C for 30 min.
5. Centrifuge at 10,000× *g* for 1 min.
6. Recover the supernatant.

Sequential incubation promotes protein digestion (56 °C) and the reduction in phenolic compounds (65 °C); this step was incorporated during protocol optimization to promote tissue lysis and protein denaturation while maintaining phenolic compounds in a reduced state through the continuous action of β-mercaptoethanol. This combination was intended to improve DNA recovery from mature coffee roots prior to silica-column purification.

#### 3.5.3. Purification Using the Quick-DNA Fecal/Soil Microbe Kits (Zymo Research, Irvine, CA, USA; Cat. No. D6010)

1. Replace the manufacturer’s step corresponding to the BashingBead™ buffer using the obtained supernatant. Follow the manufacturing protocol.
2. Store the sample at −20 °C.

### 3.6. DNA Quantification Obtained Through Different Extraction Methods

The concentration and purity of the DNA obtained by the different extraction methods were determined by spectrophotometry using a NanoDrop™ ND 2000 instrument (Thermo Scientific, Wilmington, DE, USA). Quantification was performed based on absorbance at 260 nm, while DNA quality was assessed using the A260/A280 and A260/A230 ratios.

The A260/A280 ratio was used as an indicator of protein contamination, with values close to 1.8 considered characteristic of high-purity DNA. The A260/A230 ratio was used to estimate the presence of organic contaminants and secondary compounds, such as polysaccharides and phenolic compounds, commonly associated with plant matrices.

DNA integrity was verified using electrophoresis on a 1.5% agarose gel, prepared in 1× TAE buffer. The nucleic acid stain GelRed^®^ (Biotium, Fremont, CA, USA) was added directly to the molten agarose before casting, at a final dilution of 1:10,000 (1 µL per 10 mL of gel). Electrophoresis was performed in 1× TAE running buffer. For each sample, 3 µL of DNA mixed with loading buffer was loaded. Electrophoresis was carried out at 90 V for 60 min. Subsequently, the gels were visualized under ultraviolet light to assess DNA quality by the presence of well-defined bands and the absence of degradation.

### 3.7. PCR Amplification

To verify the functional total DNA quality obtained through different extraction methods, PCR amplification of the SSU region (18S rDNA) of arbuscular mycorrhizal fungi was performed using the primers AMV4.5NF (AAGCTCGTAGTTGAATTTCG) and AMDGR (CCCAACTATCCCTATTAATCAT), expected amplicon size was approximately 300 bp.

The PCRs were prepared in a final volume of 25 µL, using 10 µL of Crystal Taq™ commercial Master Mix (2× PCR Master Mix, containing Taq DNA polymerase, dATP, dCTP, dGTP, dTTP, MgCl_2_, KCl, (NH_4_)_2_SO_4_, density reagent, and proprietary enhancing and stabilizing additive) (Jena Bioscience, Jena, Germany), 8 µL of nuclease-free water, 2.5 µL of each primer (10 µM): final primer concentration: 1 µM each and 2 µL of template DNA (adjusted to 20 ng/µL). Amplifications were performed in a thermocycler (Veriti™ Thermo Fisher Scientific Inc., Waltham, MA, USA) Under conditions previously reported for these markers: 95 °C for 45 s for denaturation, 58 °C for 45 s for hybridization, and 72 °C for 1 min for the final extension (elongation) step, for a total of 35 cycles. Amplified products were visualized using agarose gel electrophoresis under the conditions mentioned in the previous section. Each PCR run included a negative control consisting of sterile, nuclease-free water as a substitute for the template DNA, in order to verify the absence of contamination during reaction preparation.

## 4. Expected Results

The performance of the four extraction methods was first evaluated using the concentration, spectrophotometric purity, and electrophoretic integrity of the total DNA recovered from coffee roots. Because these measurements do not distinguish plant DNA from fungal or other microbial DNA, they were used only to characterize the overall yield and quality of the extracts. The suitability of each extract for AMF-targeted molecular analysis was evaluated separately by PCR amplification of the SSU 18S rDNA region using AMV4.5NF/AMDGR primers. Overall, DNA concentrations ranged from 75.5 to 6313.7 ng/µL across the four extraction methods, with considerably higher initial readings in some cases. This dispersion demonstrates significant variability between methods and samples, as well as potential overestimation due to contaminants that interfere with spectrophotometric quantification.

When comparing the evaluated methods, it was observed that modifications to the extraction protocols, particularly those incorporating predigestion and chemical treatment steps, improved consistency between replicates and increased the proportion of samples that met the minimum quality criteria (Table 1). This contrasts with methods based solely on lysis efficiency, which tended to maximize apparent concentration without ensuring adequate purity.

However, even in the samples with the highest concentrations, the A260/A230 values remained suboptimal, indicating that, despite protocol optimization, co-extraction of inhibitory compounds was not eliminated.

Method 1 produced the highest NanoDrop-derived nucleic acid concentrations, these values should be interpreted cautiously because spectrophotometric quantification measures total UV-absorbing nucleic acids and is susceptible to interference from co-extracted compounds. In terms of quality, the A260/280 values ranged from 1.15 to 1.241, while A260/230 remained consistently low (<0.9), indicating the presence of co-extracted compounds, probably of phenolic or polysaccharide nature.

In contrast, Method 2 showed a reduction in DNA concentration (from 1461.9 to 5026.4 ng/µL). A260/280 values ranged from 1.22 to 1.36, showing less dispersion and a slight increase compared to Method 1 (1.15–1.24), suggesting a partial reduction in protein contamination. However, these values remain below the optimum range (~1.8), indicating the continued presence of contaminants. Meanwhile, A260/230 values remained low (0.52–0.58) and very similar to those observed in Method 1. Method 3 yielded DNA concentrations ranging from 196.6 to 1061.7 ng/µL, which were substantially lower than those obtained with Methods 1 and 2. However, Method 3 exhibited the lowest spectrophotometric purity ratios, with A260/A280 values ranging from 1.07 to 1.11 and A260/A230 values ranging from 0.32 to 0.35, indicating significant contamination, likely from proteins, phenolic compounds, and other secondary metabolites.

On the other hand, Method 4 showed a more favorable balance between concentration and purity. Although the concentrations were moderate (75.5–302.4 ng/µL), the A260/A280 (1.84–2.05) and A260/A230 (1.37–1.71) ratios were consistently higher than those observed in the other methods. These results are consistent with reduced co-extraction and/or more effective removal of interfering compounds, suggesting that incorporating a predigestion stage with proteinase K and β-mercaptoethanol, together with the previous mechanical disruption, favors both the release and the purification of DNA in complex matrices.

DNA integrity was confirmed using agarose gel electrophoresis, which revealed well-defined, high-molecular-weight genomic bands, particularly in methods that incorporated modifications to lysis and predigestion (Figure 2), indicating the isolation of structurally intact DNA.

Method 1 showed intense signals, but these were accompanied by a strong label, suggesting the presence of partially degraded DNA or the co-extraction of compounds that affect electrophoretic migration. A similar, though less pronounced, behavior was observed in Method 2, where the bands were less intense but showed a slight improvement in definition, consistent with a lower contaminant load.

In contrast, Method 3 showed highly diffuse, poorly defined signals, with a notable accumulation of material at the bottom of the gel. This pattern is indicative of a high concentration of low-molecular-weight compounds or contaminants that interfere with proper DNA migration, which coincides with the low purity values observed spectrophotometrically. Despite the high concentrations recorded for this method, the structural quality of total DNA was compromised.

Finally, Method 4 showed more defined bands with less drag than the other methods, suggesting better DNA integrity and a lower level of contaminants. This result is consistent with the highest purity values (A260/A280 and A260/A230) obtained for this method. Overall, the results indicate that the kit extraction methods enabled the recovery of amplifiable DNA, and the modified protocols improved reproducibility and the functional quality of the DNA.

Amplification of the SSU region (18S rDNA) using the primers AMV4.5F and AMDGR allowed for the evaluation of the functional performance of the obtained DNA (Figure 3), no amplification products were observed in the negative control in any of the runs.

In Methods 1 and 2, no defined bands were observed at the expected amplicon size; the signals present corresponded mainly to low-intensity residues or possible primer dimers, and therefore were not considered positive amplifications.

In contrast Methods 3 and 4 yielded specific amplification products of the expected size (~300 bp) in samples S1–S4. This result indicates that, despite differences in spectrophotometric parameters, only the DNA obtained using methods based on the commercial kit showed adequate functional performance for PCR under the evaluated conditions Because PCR products were assessed qualitatively by agarose gel electrophoresis, no quantitative comparison of amplification efficiency between Methods 3 and 4 was performed. To objectively compare the performance of the four extraction protocols, DNA concentration and spectrophotometric quality ratios were statistically analyzed using the Friedman test, considering the four composite root samples as matched biological blocks. Significant differences among extraction methods were detected for DNA concentration (χ^2^ = 11.10, *p* = 0.011), A260/A280 ratio (χ^2^ = 12.00, *p* = 0.007), and A260/A230 ratio (χ^2^ = 12.00, *p* = 0.007). Method 1 yielded the highest DNA concentrations (4421.8 ± 1668.8 ng/µL), followed by Method 2 (3167.4 ± 1659.5 ng/µL), whereas Methods 3 and 4 produced substantially lower DNA yields (615.4 ± 356.3 and 147.6 ± 104.3 ng/µL, respectively). In contrast, Method 4 consistently exhibited the highest spectrophotometric purity, with mean A260/A280 and A260/A230 ratios of 1.90 ± 0.10 and 1.60 ± 0.16, respectively, indicating a marked reduction in co-extracted contaminants compared with the other protocols (Table 2).

## 5. Discussion

In the present study, DNA concentration alone was not a reliable indicator of extraction performance. Methods 1 and 2 produced the highest NanoDrop readings, yet neither generated specific amplification of the AMF marker. The unusually high NanoDrop concentrations obtained with Method 1 (>6000 ng/µL in some samples) most likely reflect overestimation caused by co-extracted contaminants rather than exceptionally high DNA recovery. Spectrophotometric quantification measures absorbance at 260 nm and cannot distinguish pure DNA from other UV-absorbing compounds such as phenolics, polysaccharides or degraded nucleic acids. This interpretation is supported by the low A260/A280 and A260/A230 ratios, together with the poor PCR amplification obtained with this protocol. Therefore, DNA concentration alone should not be considered a reliable indicator of extraction performance when working with phenolic-rich coffee roots.

By contrast, the modified commercial protocol yielded lower DNA concentrations but consistently produced PCR products. These observations suggest that, in coffee roots, extract purity is a better predictor of downstream amplification than DNA yield alone.

Similar observations have been reported for other plant tissues containing high concentrations of secondary metabolites, where phenolic compounds and polysaccharides interfere with DNA purification and reduce PCR performance [23]. In particular, phenolic compounds have been described as capable of oxidizing DNA and forming stable complexes with it, thereby affecting its integrity and hindering subsequent amplification [12].

This phenomenon is especially relevant in Coffea arabica, whose plant components contain high concentrations of phenolic compounds, including chlorogenic acids and other highly reactive metabolites [24,25]. In this context, the suboptimal A260/A230 values observed in this study do not necessarily indicate an extraction failure but rather reflect a limitation of the biological matrix.

The behavior of Method 1 is particularly illustrative. The phenol–chloroform-based protocol yielded relatively high concentrations but was associated with low A260/A230 values and suboptimal A260/A280 values in several samples. This pattern suggests that lysis was efficient, but that purification did not adequately remove organic compounds and inhibitors specific to the roots. This result is consistent with expectations for CTAB/phenol–chloroform protocols applied to tissues rich in secondary metabolites: they usually recover a significant amount of DNA. However, they can also carry away contaminants if additional cleaning steps or specific buffer modifications are not incorporated [26]. The review by Schenk et al. highlights precisely that the “modified” CTAB protocols differ mainly in the steps aimed at resolving problems of phenolic oxidation, polysaccharides, and secondary cleaning, which explains why CTAB that is functional in one matrix may be insufficient in another.

In Method 2, incorporating PVP into the CTAB buffer produced only a modest improvement in DNA purity. Although the A260/A280 ratio increased slightly compared with Method 1, the extracts still showed low A260/A230 values and failed to amplify the target fragment. Therefore, under the conditions evaluated here, adding PVP alone was not sufficient to overcome the inhibitory effect of compounds present in mature coffee roots [27]. However, the literature also indicates that the effect of PVP is neither universal nor necessarily sufficient on its own; its performance depends on the nature of the tissue, the concentration used, and its interactions with other components of the buffer [28]. Healey et al. reported that adding PVP can be useful for recalcitrant species, although it does not always yield usable DNA.

Method 3, corresponding to the commercial kit without pretreatment, yielded substantially lower DNA concentrations than Methods 1 and 2, with values ranging from 196.6 to 1061.7 ng/µL. However, these concentrations were associated with the worst purity indices, with A260/A280 ratios close to 1.1 and A260/A230 around 0.3. From an analytical point of view, this profile is not compatible with a clean extract, but rather with intense co-extraction of proteins, phenolic compounds, polysaccharides, or other absorbent solutes. Although it maximizes apparent material recovery, it does not improve its functional quality.

This decoupling between quantity and quality has been repeatedly observed in lignified or woody substrates [29], such as adult coffee roots, where tissue rupture releases large amounts of compounds that saturate or distort the spectrophotometric reading without improving molecular performance [30,31]. Therefore, the result of Method 3 is important because it demonstrates that the direct use of a commercial kit does not necessarily resolve the sample’s chemical complexity; in certain tissues, it may even yield an uncertain impression of success if only the concentration is considered [32].

In contrast, Method 4 showed a distinct analytical profile compared with the other extraction methods. Although it yielded the lowest DNA concentrations, it exhibited the highest post-extraction A260/A280 and A260/A230 ratios. Importantly, both Methods 3 and 4 produced the expected AMF-targeted PCR product in all four samples. Therefore, the present results do not demonstrate superior PCR efficiency for Method 4 over Method 3; rather, they show that the additional predigestion incorporated into Method 4 was associated with higher spectrophotometric ratios while maintaining successful target amplification.

This suggests that the combination of prior mechanical disruption, predigestion with proteinase K, and reductive treatment with β-mercaptoethanol improved not only DNA release but also the relative removal of contaminants [33,34]. The explanation is plausible from a chemical standpoint: proteinase K promotes the degradation of tissue-associated proteins and nucleoprotein complexes [35], while β-mercaptoethanol reduces the oxidation and polymerization of phenols and helps to destabilize disulfide-rich structures [36]. Recent literature on buffer optimization and CTAB protocols for challenging plant tissues converges on the fact that the best results are usually achieved by combining strategies of physical disruption, chemical reduction, and secondary cleanup [37], rather than relying on a single reagent or a single commercial platform.

Although the results obtained allowed for a consistent comparison of the performance of the four extraction protocols, this study has some limitations that should be considered when interpreting the findings. The samples came from a single experimental site and a small number of biological replicates; therefore, the results should be understood as a methodological evaluation of the protocols’ performance on a specific matrix of *Coffea arabica* roots, rather than as an assessment of the variability of arbuscular mycorrhizal fungal communities between sites or environmental conditions. In this regard, future studies should validate the optimized protocol using samples from different locations, coffee genotypes, and soil conditions to evaluate its robustness against greater biological variability.

Taken together, the results demonstrate that the efficiency of total DNA extraction methods from Coffea arabica roots cannot be evaluated solely by yield but must also consider the purity and functionality of the resulting genetic material in an integrated manner. Protocols that focus exclusively on maximizing cell lysis tend to overestimate the concentration due to the co-extraction of interfering compounds. In this context, the implementation of combined strategies, including mechanical disruption, enzymatic predigestion, and reductive treatment, proved crucial to improving DNA quality. Among the evaluated protocols, the modified commercial protocol yielded the highest post-extraction spectrophotometric ratios while retaining successful AMF-targeted PCR amplification in all samples, although at a substantially lower total DNA concentration than the CTAB-based methods. Both commercial kit-based protocols generated amplifiable DNA, indicating that DNA yield alone was not predictive of successful AMF-targeted PCR under the conditions evaluated.

## Figures and Tables

**Figure 1:**
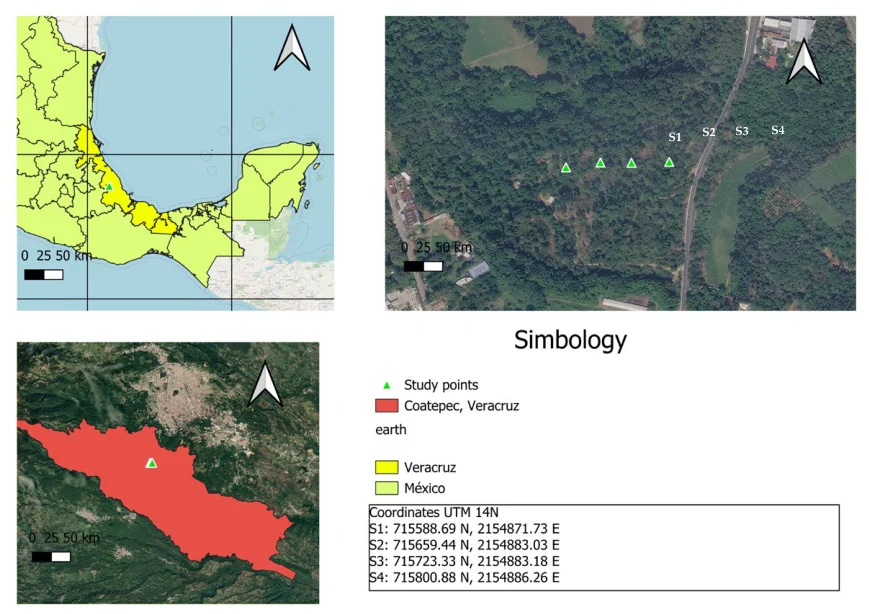
Location of the study site in Coatepec, Veracruz, Mexico. The location is shown at the national (Mexico), state (Veracruz), and local (Finca Consolapan) scales, along with the distribution of sampling points (green triangles). Sampling coordinates are presented in UTM Zone 14N. Scale bars and north arrows are shown in the corresponding map panels.

**Figure 2:**
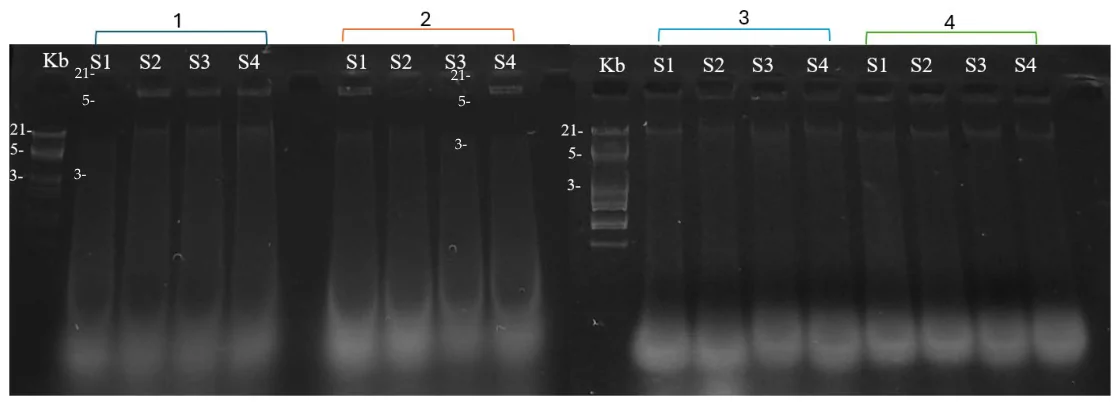
Agarose gel electrophoresis (1.5%) of genomic DNA obtained by different extraction methods (1–4). Molecular weight markers (Lambda/HindIII) are included. Differences in signal intensity indicate variations in DNA yield and quality between methods.

**Figure 3:**
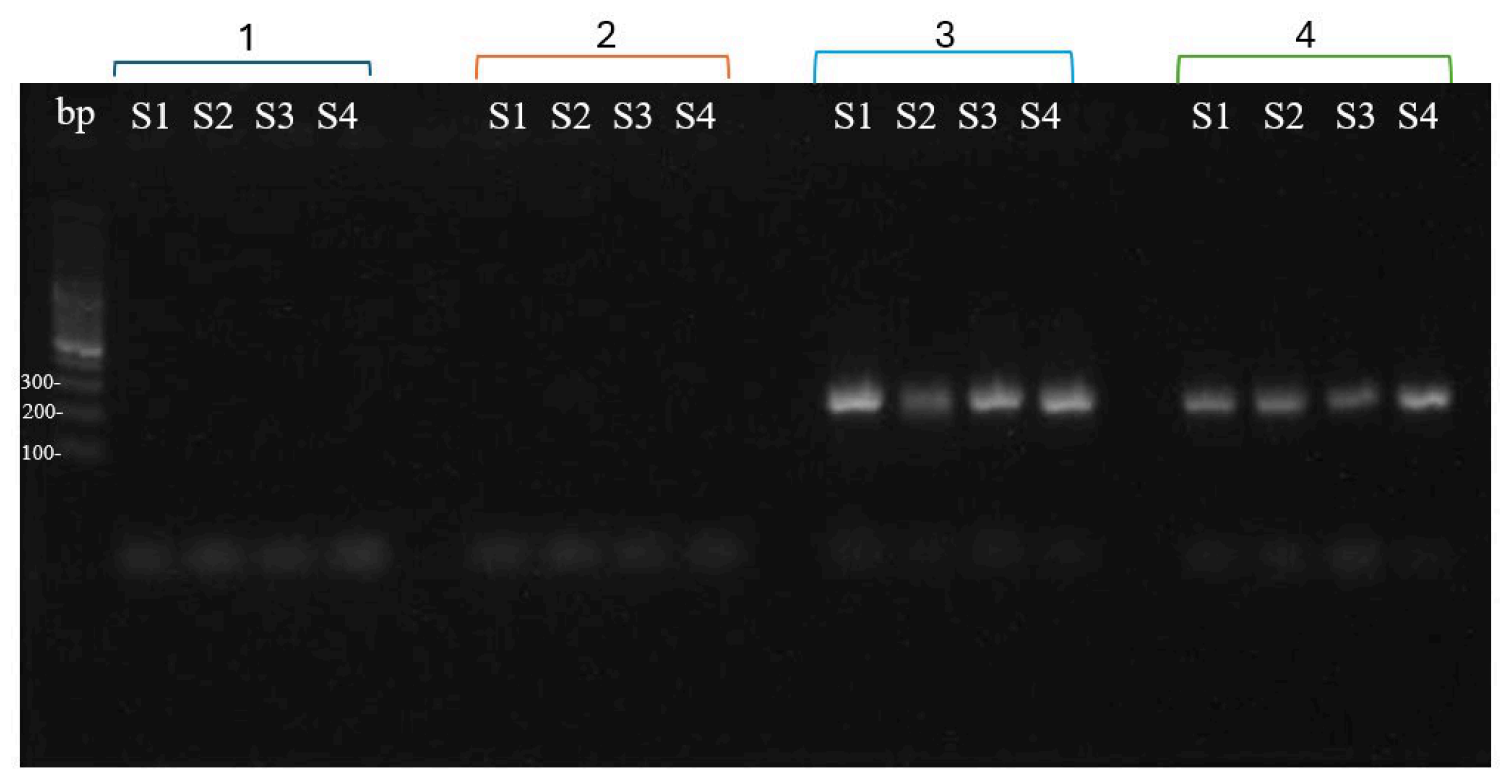
Agarose gel electrophoresis (1.5%) showing PCR amplification of the AMF SSU rDNA fragment using the primer pair AMV4.5NF/AMDGR (expected amplicon size: ~300 bp). Lane M: 100 bp DNA ladder (100–1000 bp). Lanes S1–S4 correspond to the four composite biological root samples processed with each extraction method. Methods 1–4 are indicated above the gel. Specific amplification was observed only for Methods 3 and 4, whereas Methods 1 and 2 produced only faint low-molecular-weight bands consistent with primer dimers or nonspecific products.

**Table 1:** Concentration and spectrophotometric purity parameters (A260/A280 and A260/A230) of total DNA extracted from *Coffea arabica* roots using four extraction methods. These measurements represent total root-associated nucleic acids and do not quantify AMF DNA specifically.

Method	Sample ID	DNA Concentration (ng/µL)	A260/A280	A260/A230
1	S1	4315.5	1.16	0.5
1	S2	6313.7	1.15	0.53
1	S3	2270	1.24	0.55
1	S4	4788	1.19	0.5
2	S1	5026.4	1.23	0.57
2	S2	2119.6	1.25	0.58
2	S3	1461.9	1.36	0.56
2	S4	4061.6	1.22	0.52
3	S1	547.5	1.08	0.32
3	S2	1061.7	1.11	0.35
3	S3	655.9	1.09	0.34
3	S4	196.6	1.07	0.34
4	S1	112	1.84	1.7
4	S2	100.5	1.85	1.6
4	S3	302.4	2.05	1.71
4	S4	75.5	1.87	1.37

**Table 2:** Summary statistics of DNA concentration and spectrophotometric quality obtained with each extraction method.

Method	DNA Concentration (ng/µL) Mean ± SD	CV (%)	A260/A280 Mean ± SD	CV (%)	A260/A230 Mean ± SD	CV (%)
1	4421.8 ± 1668.8	37.7	1.19 ± 0.04	3.4	0.52 ± 0.02	4.7
2	3167.4 ± 1659.5	52.4	1.27 ± 0.06	5.1	0.56 ± 0.03	4.7
3	615.4 ± 356.3	57.9	1.09 ± 0.02	1.6	0.34 ± 0.01	3.7
4	147.6 ± 104.3	70.7	1.90 ± 0.10	5.2	1.60 ± 0.16	9.9

## Data Availability

The original contributions presented in this study are included in the article. Further inquiries can be directed to the corresponding author.
